# The Antimicrobial Properties of Silver Nanoparticles in *Bacillus subtilis* Are Mediated by Released Ag^+^ Ions

**DOI:** 10.1371/journal.pone.0144306

**Published:** 2015-12-15

**Authors:** Yi-Huang Hsueh, Kuen-Song Lin, Wan-Ju Ke, Chien-Te Hsieh, Chao-Lung Chiang, Dong-Ying Tzou, Shih-Tung Liu

**Affiliations:** 1 Graduate School of Biotechnology and Bioengineering, Yuan Ze University, Taoyuan, Taiwan; 2 Department of Chemical Engineering and Materials Science, Yuan Ze University, Taoyuan, Taiwan; 3 Graduate Institute of Biomedical Sciences, and Research Center for Bacterial Pathogenesis, Chang Gung University, Taoyuan, Taiwan; Institute for Materials Science, GERMANY

## Abstract

The superior antimicrobial properties of silver nanoparticles (Ag NPs) are well-documented, but the exact mechanisms underlying Ag-NP microbial toxicity remain the subject of intense debate. Here, we show that Ag-NP concentrations as low as 10 ppm exert significant toxicity against *Bacillus subtilis*, a beneficial bacterium ubiquitous in the soil. Growth arrest and chromosomal DNA degradation were observed, and flow cytometric quantification of propidium iodide (PI) staining also revealed that Ag-NP concentrations of 25 ppm and above increased membrane permeability. RedoxSensor content analysis and P_hag_-GFP expression analysis further indicated that reductase activity and cytosolic protein expression decreased in *B*. *subtilis* cells treated with 10–50 ppm of Ag NPs. We conducted X-ray absorption near-edge structure (XANES) and extended X-ray absorption fine structure (EXAFS) analyses to directly clarify the valence and fine structure of Ag atoms in *B*. *subtilis* cells placed in contact with Ag NPs. The results confirmed the Ag species in Ag NP-treated *B*. *subtilis* cells as Ag_2_O, indicating that Ag-NP toxicity is likely mediated by released Ag^+^ ions from Ag NPs, which penetrate bacterial cells and are subsequently oxidized intracellularly to Ag_2_O. These findings provide conclusive evidence for the role of Ag^+^ ions in Ag-NP microbial toxicity, and suggest that the impact of inappropriately disposed Ag NPs to soil and water ecosystems may warrant further investigation.

## Introduction

Silver nanoparticles (Ag NPs) are the most widely used nanomaterial in healthcare today, with total annual worldwide production estimated to be in the range of 500 tons [1]. The superior antimicrobial, antifungal, and antiviral properties of Ag NPs mean that they are frequently present in coatings for bone prostheses, surgical devices, infusion systems, and dental composites [2–4], as well as air/water filters, food containers, textiles, and many other consumer products [5–7]. Ag NPs are known to possess oligodynamic properties, and can kill antibiotic-resistant microbes while exerting limited cytotoxicity against mammalian cells [8–11]. However, the mechanisms underlying Ag-NP microbial toxicity remain the subject of intense debate.

Dissolved Ag^+^ ion concentrations have been found to dictate the toxicity of Ag NPs [12–13], with nanosilver serving as a source for Ag^+^ ions [14–15]. A recent study further showed that Ag NPs leach Ag^+^ ions under aerobic but not anaerobic conditions, while Ag-NP microbial toxicity was also limited to aerobic conditions [16]. Positive surface charge has also been found to protect *Bacillus* species bacteria against Ag-NP toxicity [17–18]. These studies offer indirect evidence for the role of Ag^+^ ions in Ag-NP antimicrobial activity.

It is known that Ag^+^ ions can rupture microbial cell walls, denature cellular proteins, block cell respiration, and eventually induce cell death [19–21], but it has been posited that Ag-NP toxicity cannot be solely attributed to release Ag^+^ ions, and bacterial contact with nanosilver particles [22] has been proposed to be a key factor for Ag-NP toxicity induction. It has also been suggested that the method of synthesis, size [23], shape, or surface coating of Ag NPs can affect toxicity [24–25]. Ag-NPs can be synthesized by physical methods such as evaporation-condensation and laser ablation; chemical methods such as reduction by organic or inorganic agents, photo-induced reduction, microemulsion techniques, irradiation, electrochemical/sonoelectrochemical synthesis, and microwave-assisted synthesis; and bio-based methods utilizing bacteria, fungi, algae, or plants [26–27]. The mode of synthesis can determine the final size, shape, and chemical composition of Ag NPs, which will then affect Ag-NP plasmonic properties to potentially influence antimicrobial activity [28–31]. Commercially available Ag NPs differ broadly in terms of size, morphology, and degree of agglomeration [32], and evidence suggests that smaller Ag-NPs may exert greater toxicity [33–35]. In the case of *Bacillus subtilis*, Ag NPs of around 50 nm in diameter were found to induce a maximum log reduction of 2 in bacterial cell numbers at concentrations of 100 ppm [36], while the minimum inhibitory concentration (MIC) of 3-nm Ag NPs was just 40 ppm [37].

In this study, we examined the effects of Ag NPs in *B*. *subtilis*, and assessed the mechanisms underlying Ag-NP toxicity via X-ray absorption spectroscopy (XAS). *B*. *subtilis* is a ubiquitous soil bacterium known to colonize the surface of plant roots as a biofilm, produce various functional lipopeptides, induce plant immunity against a range of diseases, and support the natural rhizosphere surrounding plant roots [38–41]. With the increased use of Ag NPs, the risk of contamination from improper processing or disposal is heightened, and therefore it is important to understand how Ag NPs may affect soil and water microorganisms to the detriment of local ecosystems. For example, Ag NPs from everyday products can enter the waste stream and become concentrated in sludge during the wastewater treatment process [42], and the US Environmental Protection Agency has reported Ag concentrations ranging from 1.94 to 856 mg/kg in sludge samples (US EPA 822-R-08-014, January 2009. http://water.epa.gov/scitech/wastetech/biosolids/tnsss-overview.cfm). A large proportion of biosolids from sludge will be dried and applied as fertilizer to agricultural soil [42], thereby increasing the risk of Ag-NP dissemination into the environment. A recent field scenario study showed that the application of a single 0.14 mg Ag/kg soil dose of Ag NPs via sewage biosolids significantly affected the composition of soil bacterial communities, reduced microbial biomass by 35%, and inhibited the activities of microbial extracellular enzymes [43]. Sublethal levels of Ag NPs have also been found to trigger quorum-sensing responses in *B*. *subtilis*, with varying effects on cell and ecosystem viability [44]. We sought to ascertain the effects of comparable or higher Ag-NP doses upon *B*. *subtilis*, and further used XAS to examine the characteristics of Ag atoms in Ag NP-treated *B*. *subtilis* cells. XAS is an excellent tool for short-range order characterization of the valence and local structure of Ag species (metallic Ag or ionic Ag^+^) within *B*. *subtilis* cells [45]. X-ray absorption near edge structure (XANES) spectroscopy can provide information on the electronic configuration, stereochemistry, and oxidation state of Ag atoms [46–47], and extended X-ray absorption fine structure (EXAFS) spectroscopy can offer additional information regarding the atomic arrangement of Ag atoms, in terms of bond distance, coordination number, near-neighbor type, and thermal/static disorder [45, 48]. Therefore, XANES and EXAFS analysis can elucidate the oxidation state and fine structure of Ag atoms in *B*. *subtilis* cells placed in contact with nanosilver, which would help to clarify Ag-NP microbial toxicity mechanisms.

## Materials and Methods

### Ag-NP synthesis and characterization

Silver nitrate (AgNO_3_, 0.85 g) and polyvinylpyrrolidone (PVP, 1.0 g) were dissolved in ethylene glycol (EG, 100 mL), with continuous stirring until a homogeneous solution was obtained. PVP was used here to stabilize the Ag suspension. After solution mixing, pulse microwave-assisted synthesis was conducted as follows: the Ag-containing solution was placed in the center of a household microwave oven (Tatung Co., 900 W, 2.45 GHz, Taiwan) in which one thermocouple had been equipped to detect reaction temperature. Pulse microwave-assisted synthesis was carried out at 40°C under a direct current power supply (8 V, 90 A, 720 W).

Microwave-assisted synthesis has been reported to produce consistently small Ag NPs that are uniform and stable at room temperature [26]. We proceeded to characterize our prepared Ag-NP samples in terms of structure, morphology, and elemental composition, using field emission scanning electron microscopy (FE-SEM), transmission electron microscopy (TEM), and X-ray diffraction (XRD). FE-SEM was conducted with a JEOL JSM-6701F field emission scanning electron microscope, to determine the morphology, microstructure, and particle size distribution of synthesized Ag NPs. TEM images were collected with a JEOL TEM-2010 scanning electron microscope. A single 20-μL drop of the silver nanoparticle suspension was placed on 400-mesh TEM grids with a carbon support film (Agar Scientific, Essex, UK). The dried preparations were rinsed with ethanol and dried again, before being mounted on the appropriate holder and placed in the microscope. Transmitted electron images were collected in bright field mode at an accelerating voltage of 200 keV, with the specimen set at an 8 mm working distance. Samples were analyzed within 1 hr to avoid shape degradation. We subsequently used Gatan DigitalMicrograph software to analyze representative TEM images, in order to determine the size of synthesized Ag NPs. A total of 100 nanoparticles were randomly selected for size assessment. The solid phase microstructure and crystallinity of Ag NPs was then characterized by XRD at a scanning range of 20–80° (2θ) and a scan rate of 4° (2θ)/min on a Rigaku RU-H3R diffractometer, using monochromatic Cu K_α_ radiation with a wavelength of 1.5405 Å at 30 kV and 20 mA.

### *B*. *subtilis* growth conditions and assessment of Ag-NP antibacterial effects

*B*. *subtilis* wild-type strain 3610 [49] was maintained at 37°C in Luria-Bertani (LB; 10 g tryptone, 5 g yeast extract, 5 g NaCl per liter) broth or on 1.5% Bacto agar plates. Ag-NPs were added where appropriate to achieve the following final concentrations: 0, 1, 5, 10, 25, or 50 ppm. For growth assays conducted in a minimal medium [50], overnight cultures were diluted 100-fold in the minimal medium and grown at 37°C under shaking at 200 rpm for 12 h, with Ag NPs added where appropriate to achieve the following final concentrations: 0, 0.1, 1, or 10 ppm.

To assay time-dependent growth inhibition caused by Ag NPs, overnight cultures of approximately 1 × 10^9^ CFU/mL were diluted 100-fold into 50 mL of LB broth in 250 mL flasks. Ag-NPs were added where appropriate, to achieve final concentrations of 0, 1, 5, 10, 25, or 50 ppm. Cultures were then grown for up to 30 h at 37°C, with shaking at 200 rpm. Bacterial growth was measured by optical density at 600 nm (OD_600_). To assess the antibacterial effects of Ag NPs against *B*. *subtilis*, initial cultures (1 × 10^9^ CFU/mL) were prepared from 50-mL LB liquid cultures harvested at exponential growth. Bacterial cells were treated with Ag NPs at increasing concentrations of 0–50 ppm for 5 h, with shaking at 200 rpm. Both treated and untreated cultures were then serially diluted and plated on LB agar plates at the time points indicated. Plates were incubated overnight at 37°C and then subjected to a colony count [51]. All experiments were performed in triplicate and averaged, and statistical analysis indicated the results were of good reliability.

### Chromosomal DNA integrity analysis

Chromosomal DNA was isolated from *B*. *subtilis* cells grown in LB medium with final concentrations of 0, 1, 5, 10, 25, or 50 ppm of Ag NPs, using a Wizard Genomic DNA Purification kit (Promega, Madison, WI, USA). Each lane was loaded with ~1 μg DNA, and gel electrophoresis was performed on a 1.0% agarose gel for 30 min at 120 V.

### Transmission electron microscopy (TEM) of bacterial cells

Bacterial cells were fixed in 2% v/v paraformaldehyde/3% v/v glutaraldehyde in 0.1 M cacodylate buffer (pH 7.4) for 2 h at 4°C. Samples were then washed in 0.1 M cacodylate buffer three times, and postfixed in 1% osmium tetroxide at 4°C for 1 h. Bacterial cells were then washed three times as described above, dehydrated in a graded series of ethanol, and embedded in Eponate 12 resin (Ted Pella Inc., Redding, CA) for 7 h. Sections of 70–80 nm in thickness were sliced and stained with uranyl acetate and lead citrate, then viewed under a Hitachi transmission electron microscope (H-7500, Tokyo, Japan) at 200 kV. TEM montage images were manually acquired at 75,000× magnification.

### Measurement of RedoxSensor activity

RedoxSensor activities of *B*. *subilis* 3610 were determined using a BacLight^TM^ RedoxSensor^TM^ Green Vitality Kit (Molecular Probes, Eugene, OR, USA). Overnight cultures of *B*. *subtilis* 3610 were treated with the indicated concentrations of Ag-NPs for 3 h at 37°C. Cells were then washed and diluted 10-fold in 1× PBS buffer, then mixed with 1 μL of RedoxSensor^TM^ Green reagent and vortexed. To assess membrane integrity, 1 μL of propidium iodide (PI) was added, and the mixture was incubated in the dark at room temperature for 5 min. Stained cells (10 μL) were spotted onto a clean slide and covered with a poly-L-lysine treated coverslip. Slides were observed under a Leica TCS-SP2 laser-scanning confocal microscope at a magnification of 630×.

### Assessing Ag-NP impact on P_hag_-GFP expression

Plasmid pHag-gfp was constructed by inserting a DNA fragment containing the gfp sequence transcribed from the hag promoter into pHY300PLK (Takara, Shiga, Japan). *B*. *subtilis* 3610(P_hag_-GFP) was treated with Ag NPs for 3 h at 37°C. Bacterial cultures (3 mL) were centrifuged and washed with 300 μL 1× T-Base buffer [15 mM (NH_4_)_2_SO_4_, 80 mM K_2_HPO_4_, 44 mM KH_2_PO_4_, 3.4 mM sodium citrate, and 3 mM MgSO_4_], and bacterial cells were resuspended in 50 μL 1× T-Base buffer containing 10 μg/mL FM1-43FX and 5 μg/mL DAPI. The mixture was incubated in the dark at room temperature for 15 min, and 4 μL of stained cells were spotted onto a clean slide and covered with a poly-L-lysine treated coverslip. Slides were observed under a Leica TCS-SP2 laser-scanning confocal microscope at a magnification of 3,150×.

### Flow cytometry analysis of P_hag_-GFP expression and RedoxSensor activity

For the P_hag_-GFP expression assay, bacterial cultures were treated with indicated concentrations of Ag NPs for 3 h at 37°C, after which 3 mL of culture was centrifuged and washed with 300 μL of 1× T-Base buffer. Cells were resuspended in 1 mL of 1× T-Base buffer, and flow cytometry was directly performed on a FACSCalibur flow cytometer (BD Biosciences, San Jose, CA, USA). Fluorescence filters and detectors were all standardized, with green fluorescence collected in the FL1 channel (530 ± 15 nm). All parameters were collected as logarithmic signals. For the RedoxSensor activity assay, bacterial cells were treated with the indicated concentrations of Ag NPs, washed and diluted 10-fold in 1× PBS buffer, then mixed with 1 μL of a 1:10 dilution of RedoxSensor^TM^ Green reagent and vortexed. To assess membrane integrity, 1 μL of a 1:10 dilution of propidium iodide (PI) was added, and the mixture was incubated in the dark at room temperature for 5 min. Samples (1 mL) were assayed by flow cytometry using a FACSCalibur flow cytometer and standardized fluorescence filters and detectors. Green fluorescence was collected in the FL1 channel (530 ± 15 nm), and red fluorescence was collected in the FL3 channel (> 650 nm). All parameters were collected as logarithmic signals. Data were analyzed using CellQuest Pro software. In density plots of light scatter properties, bacterial cells were gated from irrelevant counts for fluorescence analyses. Flow cytometry was calibrated using BD Calibrite beads (BD Biosciences, San Jose, CA, USA). Data are representative of results derived from two separate experiments.

### XANES and EXAFS analyses

XANES and EXAFS results were collected at the Wiggler beamline 01C1 in the National Synchrotron Radiation Research Center (NSRRC) of Taiwan. The electron storage ring was operated at an energy level of 1.5 GeV and a current of 100–200 mA. An Si(111) DCM was used for providing highly monochromatized photon beams with an energy of 6–33 keV (BL01C1) and resolving power (E/△E) of up to 7,000. Data were collected in fluorescence or transmission mode with a Lytle ionization detector [48] for Ag (25,514 eV) K-edge experiments at room temperature. Photon energy was calibrated by characteristic pre-edge peaks in the absorption spectra of metallic silver standards. Local structural parameters, such as the bond length (R), coordination number (CN), and Debye-Waller factor (σ) for different coordination shells surrounding the absorbing atoms, were obtained through non-linear least-square fit. Raw absorption data in the region of 50–200 eV below the edge position were also fit to a straight line, using least-square algorithms. XANES analysis was extended to energy levels of the order of 50 eV above the edge. Spectra were measured with a step size equivalent to less than 0.5 eV in the near-edge, and with a count time weighted to be proportional to *k* ^3^ at high energy. Data were normalized using the program Athena (VI), with a linear pre-edge and polynomial post-edge background subtracted from the raw ln(*I*_*t*_ */I*_*0*_) data, and then analyzed using the Artemis (VI) software, which makes use of the FFEF code-8 [52–54]. After calibration, samples were background-corrected, using a linear pre-edge region and a polynomial for the post-edge region, and subsequently normalized. EXAFS energy spectra were then converted to wavevector *K* space. The resulting scatter curve was weighed by *K* ^3^ to enhance dampened scattering oscillations. This curve was followed by Fourier transformation to yield the radial structure function [54]. These data directly reflect the average local environment around the absorption atoms. Spectra were analyzed using the software package IFEFFIT [52–53]. The theoretical paths for Ag–Ag, Ag–S, and Ag-O species used for fitting the first coordination shell of the experimental data were generated using the FEFF-8 program, based on the crystallographic data of individual species [52–53]. The coordination number, interatomic distance, Debye-Waller factor, and inner potential correction were used as variable parameters for the fitting procedures. In the case of XANES spectra, the intensity of the pre-edge peak, centered at 25,514 eV and present in the Ag K-edge spectra of the different samples, was used to estimate the relative amount of Ag^0^ or Ag^+^ species.

## Results

### Ag NPs induce growth inhibition and chromosomal DNA degradation in *B*. *subtilis*

**Fig 1:**
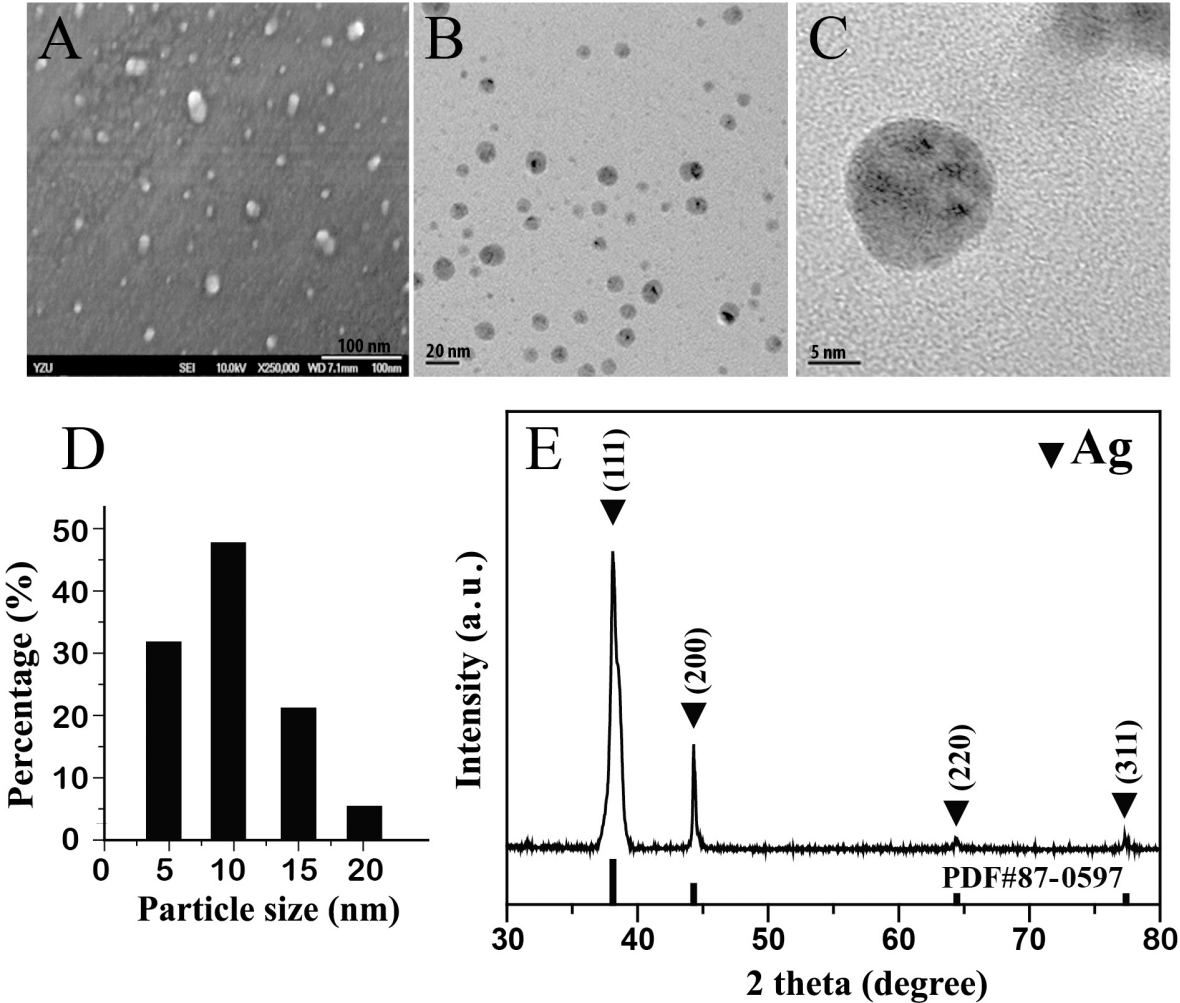
Morphology, particle size, and crystal structure characterization of Ag-NPs. (A) SEM images of Ag NPs used in this study; white bar: 100 nm. (B) TEM images of Ag NPs used in this study; scale bar: 20 nm. (C) TEM image of a representative Ag NP from those used in this study; scale bar: 5 nm. (D) Size distribution histograms of Ag NPs derived from SEM analysis. (E) XRD patterns of synthesized Ag NPs.

Ag NPs can vary broadly in terms of size and morphology [32], and as Ag-NP size has been cited as a determining factor in toxicity [23], it is important to assess the diameter and structure of Ag-NPs used in toxicity experiments, in order to allow for meaningful comparison across studies. Ag NPs used in this study were derived via pulse microwave-assisted synthesis and characterized by FE-SEM, XRD, and TEM. FE-SEM micrographs revealed the average particle diameter of Ag NPs to be about 10 nm (Fig 1A and 1D). TEM images corroborated these results (Fig 1B and 1C), and a random count of 100 Ag NPs revealed the mean particle size to be about 10.9654 nm. XRD patterns showed four main characteristic diffraction peaks, respectively at [111], [200], [220], and [311] (Fig 1E), which correspond to 2θ = 38.4, 43.3, 64.6, and 77.7, based on the band for face-centered cubic (FCC) structures of silver (JCPDS Card Number 87–0597). No peaks were observed in any other phases, indicating that single-phase Ag NPs with a cubic structure were obtained. The overall pattern was comparable with XRD spectra previously reported for such Ag-NPs in other studies [55].

**Fig 2:**
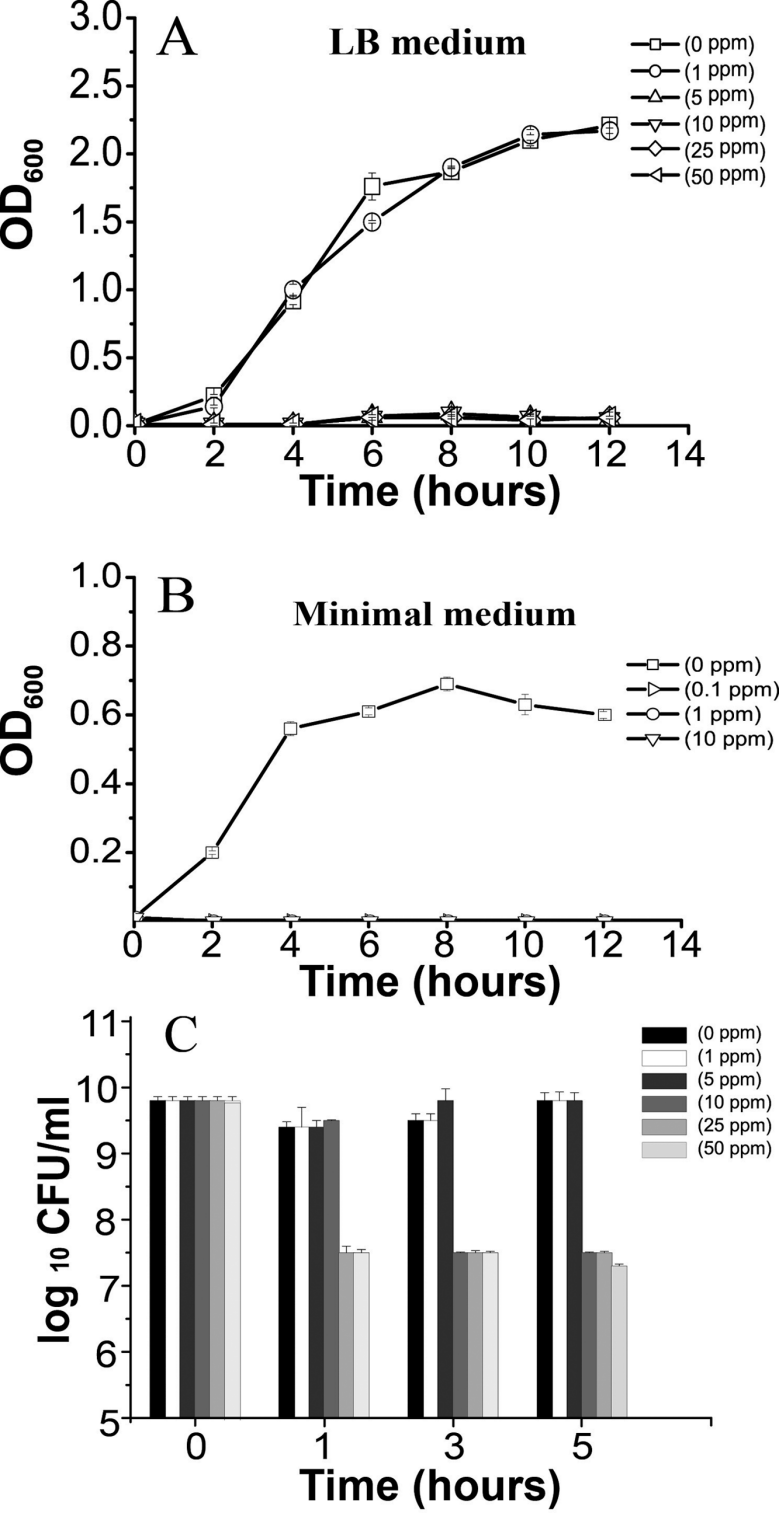
Effects of various Ag-NP concentrations on *B*. *subtilis* growth. (A) Growth analysis curves of *B*. *subtilis* in rich media treated with Ag NPs, measured by monitoring OD_600_ values. (B) Growth analysis curves of *B*. *subtilis* in minimal media treated with Ag NPs. Ag-NP concentrations are shown as -▢-: 0 ppm; -▷-: 0.1 ppm; -○-: 1 ppm; -△-: 5 ppm; -▽-: 10 ppm; -◇-: 25 ppm; and -◅-: 50 ppm. (C) Ag-NP antibacterial activity against *B*. *subtilis* cells.

*B*. *subtilis* is one of the best-characterized model organisms for Gram-positive bacteria, and is ubiquitously present in soil and water ecosystems, where it is known to exert a broad range of beneficial effects [37–41, 56]. We therefore sought to assess the effects of Ag NPs upon *B*. *subtilis*, in order to elucidate the toxicity mechanisms involved and to gauge the potential impact of Ag-NP exposure. We treated *B*. *subtilis* 3610 cultures with 0–50 ppm of Ag NPs, and evaluated bacterial growth over a period of 12 h in a rich LB medium. At concentrations > 5 ppm, Ag NPs inhibited bacterial growth for 12 h (Fig 2A), and lethal effects were observed at Ag-NP concentrations ≥ 10 ppm. The effect of Ag NPs was also tested on *B*. *subtilis* cells grown in a low nutrient medium, and it was found that 0.1 ppm of Ag NPs was sufficient to halt cell growth for over 12 h (Fig 2B). These results are in line with the general understanding that cultures in minimal medium are more sensitive to Ag NPs, compared with cultures in rich medium. We further added Ag NPs to approximately 1 × 10^9^ CFU/mL of freshly grown *B*. *subtilis* cultures, and incubated the mixtures for 5 h. Treatment with 25 or 50 ppm of Ag NPs led to a 2 log reduction of colony-forming units after 1 h of incubation, while treatment with 10 ppm of Ag NPs achieved a similar result after 3 h of incubation (Fig 2C), thus confirming that Ag NPs can hinder *B*. *subtilis* growth and entry to the exponential phase; moreover, significant lethality was observed with Ag-NP concentrations ≥ 10 ppm (Fig 2C).

Previous *in vitro* research has shown that Ag NPs are capable of inducing strand breaks in isolated plasmid DNA [57]; however, few *in vivo* studies examining the effect of Ag NPs on chromosomal DNA are available. We therefore used agarose gel electrophoresis to examine the chromosomal DNA integrity of *B*. *subtilis* cells treated with 0–50 ppm concentrations of Ag NPs. DNA electrophoresis on a 1.0% agarose gel revealed significant degradations in the chromosomal DNA of *B*. *subtilis* cells treated with 10–50 ppm of Ag NPs (Figure A in S1 File), suggesting that Ag-NP concentrations of 10 ppm or greater are sufficient to compromise chromosomal DNA integrity in bacterial cells. We further employed TEM to observe the morphological changes in *B*. *subtilis* cells following Ag-NP treatment, and found that bacterial chromosomal DNA became looser at Ag-NP concentrations greater than 10 ppm (Figure B in S1 File), suggesting that chromosomal DNA integrity and cell morphology may be adversely affected by Ag-NP concentrations as low as 10 ppm.

### Ag NPs reduced *B*. *subtilis* RedoxSensor activity and P_hag_-GFP expression

**Fig 3:**
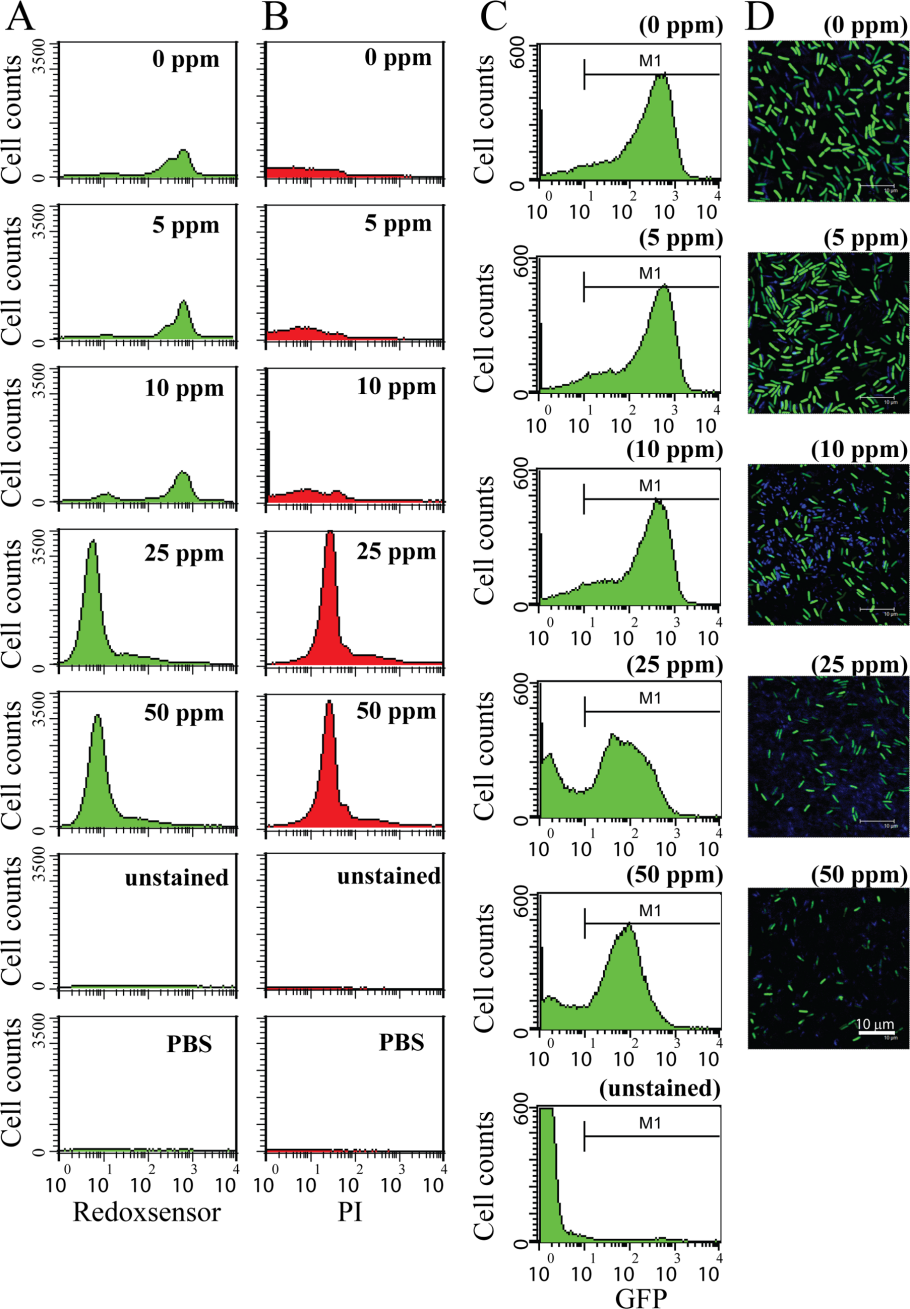
Analysis of RedoxSensor content, PI staining, and P_hag_-GFP expression in *B*. *subtilis*. (A) Wild-type bacteria were grown for 3 h with Ag-NP concentrations of 0, 5, 10, 25, or 50 ppm, and then subjected to RedoxSensor^TM^ Green (green) and (B) PI (red) staining. PBS buffer-only and unstained controls were provided. The X axis indicates RedoxSensor or PI fluorescence intensity (arbitrary units: au), as measured by flow cytometry, and the Y axis indicates cell counts. The flow cytometry data is representative of two separate experiments. RedoxSensor activity presents a false green color, and PI presents a false red color. Scale bar: 10 μm. (C) *B*. *subtilis* P_hag_-GFP expression after treatment with varying concentrations of Ag NPs. Unstained controls were included. The X axis indicates GFP fluorescence intensity (arbitrary units: au), and the Y axis indicates cell counts. Flow cytometry data is representative of two separate experiments. (D) Fluorescent micrographs indicate P_hag_-GFP expression in *B*. *subtilis* cells cultivated with various concentrations of Ag NPs for 3 h. GFP reporter expression presents as a false green color. DAPI presents a false blue color. Scale bar: 10 μm.

Ag NPs have been said to increase reactive oxygen species (ROS) levels in cells [21], and thus we sought to examine whether reductase activity in *B*. *subtilis* was affected by Ag NPs. Cells were grown to early stationary phase at approximately 10^9^ CFU/mL, then treated with 0–50 ppm of Ag NPs. After 3 h of treatment, cells were washed, and approximately 10^9^ CFUs were subjected to staining with RedoxSensor Green^TM^, a fluorescent dye that yields green fluorescence (488 nm excitation) when modified by reductases [58], and PI (red), to assess reductase activity and membrane integrity. RedoxSensor activity was quantified using flow cytometry, and results showed that treatment with Ag NPs led to general declines in the geometric mean of reductase activity levels (0 ppm: 390.89; 5 ppm: 576.67; 10 ppm: 564.54; 25 ppm: 63.13; 50 ppm: 66.81) and the percentage of gated cells in the total cell population (0 ppm: 90.38%; 5 ppm: 92.71%; 10 ppm: 76.59%; 25 ppm: 0.03%; 50 ppm: 0.05%), with significant decreases observed following treatment with ≥ 25 ppm of Ag NPs (Fig 3A). Furthermore, Ag-NP concentrations above 25 ppm were found to affect the integrity of cell membranes after just 3 h of treatment, with dramatic increases seen in the percentage of cells exhibiting PI fluorescence (0 ppm: 8.18%; 5 ppm: 6.22%; 10 ppm: 17.79%; 25 ppm: 89.51%; 50 ppm: 90.52%), indicating increased membrane permeability (Fig 3B). Together, these results show that after treatment with 25 or 50 ppm of Ag NPs, reductase activity decreased significantly in *B*. *subtilis* cells, while membrane permeability increased significantly (Fig 3A and 3B).

We next sought to ascertain the effect of Ag NPs on protein expression. In a separate experiment, *B*. *subtilis* 3610 (P_hag_-GFP) was grown until early stationary phase, at approximately 10^9^ CFU/mL, and treated with varying concentrations of Ag-NPs for 3 h. P_hag_-GFP expression was then quantified using flow cytometry. Results showed that both levels of P_hag_-GFP expression (0 ppm: 241.81; 5 ppm: 255.96; 10 ppm: 205.19; 25 ppm: 73.94; 50 ppm: 67.91) and the percentage of gated cells (0 ppm: 93.58%; 5 ppm: 93.9%; 10 ppm: 90.22%; 25 ppm: 62.17%; 50 ppm: 74.3%) decreased significantly after treatment with ≥ 25 ppm of Ag NPs (Fig 3C). These results were further confirmed with fluorescent micrographs (Fig 3D), which mirrored the flow cytometry findings. This suggests that Ag-NP concentrations of 25 ppm and above can adversely impact P_hag_-GFP and cytosolic protein expression in *B*. *subtilis* cells.

### XANES/EXAFS analysis of Ag-NP fine structure in *B*. *subtilis* cells

**Fig 4:**
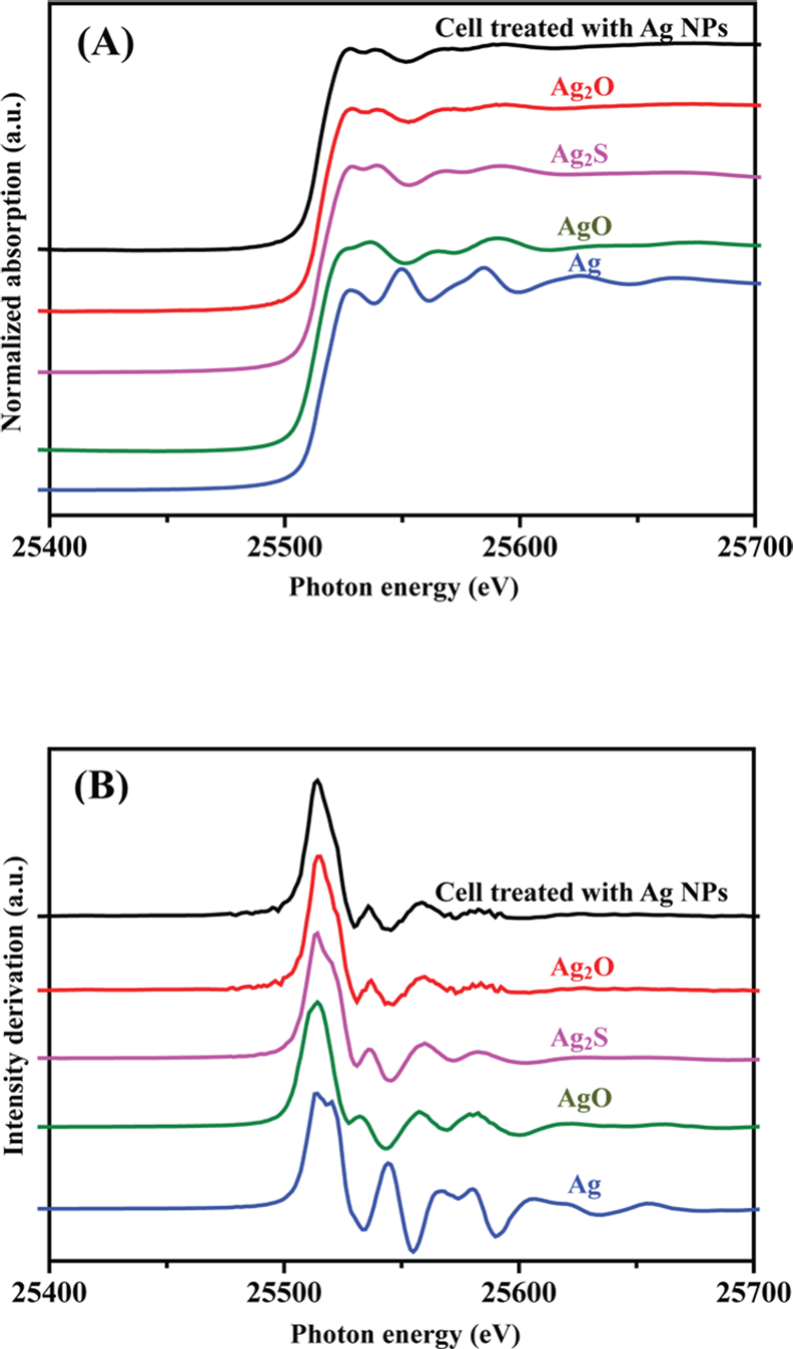
K-edge XANES spectra of silver standards and Ag NP-treated *B*. *subtilis* cells. (A) Normalized Ag K-edge XANES spectra. (B) Derivative Ag K-edge XANES spectra. The spectra are displaced vertically for clarity.

The question of whether Ag NPs exert antimicrobial effects through inherent toxicity or the release of Ag^+^ ions is yet to be resolved. We therefore wished to characterize the oxidation state and fine structure of Ag atoms in *B*. *subtilis* cells treated with Ag NPs, using XANES and EXAFS. Near-edge structure spectra can provide information on Ag valence states, and can be fitted to spectra derived from established standards to determine the oxidation state of Ag atoms in *B*. *subtilis* cells treated with Ag NPs. The normalized silver K-edge spectra of *B*. *subtilis* cells treated with 100 ppm of Ag NPs, as well as spectra from silver standards (Ag, AgO, Ag_2_O, and Ag_2_S) are presented in Fig 4A, while derivative spectra are shown in Fig 4B. Several sharp absorption peaks in the range between 25,500 and 25,650 eV were observed in the silver standards. At 25,528 eV, spectra derived from *B*. *subtilis* cells treated with 100 ppm of Ag NPs overlapped with Ag, AgO, Ag_2_O, and Ag_2_S standards. However, at 25,538 and 25,569 eV, patterns only fit the Ag_2_O and Ag_2_S standards (Fig 4A and 4B). The pattern at 25,590 eV only fit the Ag_2_O standard, indicating that the Ag species in Ag NP-treated *B*. *subtilis* cells is very likely to be Ag_2_O (Fig 4B).

**Fig 5:**
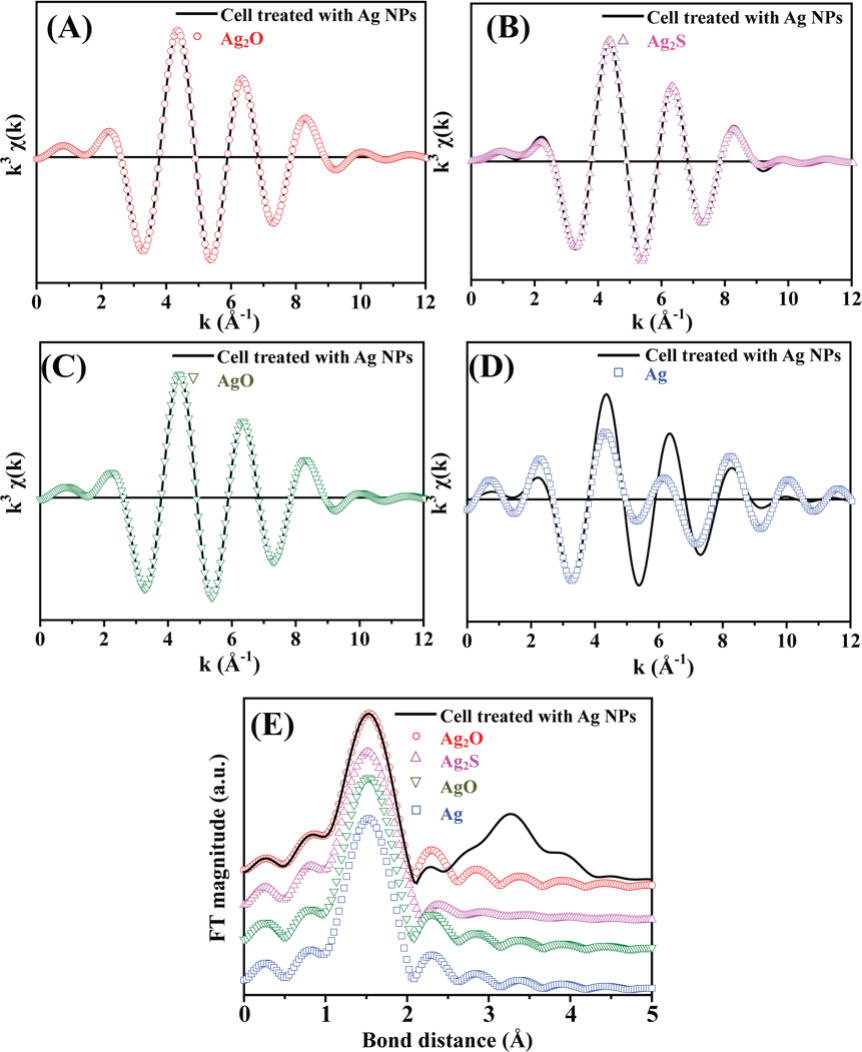
K-edge EXAFS oscillation k^3^χ(k) of silver standards and Ag NP-treated *B*. *subtilis* cells. Spectra from Ag NP-treated *B*. *subtilis* cells were fitted to spectra from (A) Ag_2_O, (B) Ag_2_S, (C) AgO, and (D) Ag standards. (E) Ag Fourier transformation (FT) spectra of silver standards and *B*. *subtilis* cells treated with 100 ppm of Ag NPs.

EXAFS analysis has elemental specificity and is highly sensitive, and therefore can be used to determine the chemical states of target species that are present at very low concentrations. We used EXAFS to analyze the types of neighbors, bond length (R), and coordination number (CN) of silver atoms in *B*. *subtilis* cells treated with 100 ppm of Ag NPs. Fig 5A–5D shows the k^3^-weighted and least-square fitted Ag K-edge EXAFS inverse Fourier transformation oscillation spectra from silver standards (Ag, AgO, Ag_2_O, and Ag_2_S) and *B*. *subtilis* cells treated with 100 ppm of Ag NPs. Contributions from the electronic scatter effect between Ag atoms in the first electron shell were ascertained through inverse Fourier transformation of EXAFS spectra between 1 to 2 Å, and the results showed that spectra from the silver standards, Ag_2_O (Fig 5A), Ag_2_S (Fig 5B), and AgO (Fig 5C), closely fitted spectra derived from Ag NP-treated *B*. *subtilis* cells. Further analysis revealed that the bond distance spectra of Ag NP-treated *B*. *subtilis* cells (black) clearly overlapped with Ag_2_O (red) at 1.55 Å (Fig 5E), thus confirming the coordination shell as Ag(I)-O. Moreover, an elemental analysis of Ag NP-treated *B*. *subtilis* cells indicated that only limited levels of sulfur (S) were present (Table C in S1 File), thereby excluding the possibility of the silver species being primarily present as Ag_2_S. Quantitative analysis showed that the bond length and coordination number of Ag_2_O in *B*. *subtilis* cells treated with Ag NPs was 2.06 Å and 1.00, respectively; and R-factor analysis further confirmed the precision of EXAFS model fitting (Ag_2_O < AgO < Ag_2_S < Ag, with lower R-factors indicating tighter fits) (Table 1). These results show that Ag-NP microbial toxicity likely stems from Ag^+^ ions released from Ag NPs (Ag^0^), which can enter *B*. *subtilis* cells to exert toxic effects, and subsequently become oxidized intracellularly to become Ag_2_O.

**Table 1:** Fine structural parameters of cells treated with 100 ppm of Ag-NPs analyzed by using EXAFS spectra. CN^a^: Coordination number; R^b^: Bond distance; σ^c^: Debye-Waller factor.

Sample	Shell (1^st^)	CN^a^ (± 0.05 Å)	R (Å)^b^ (± 0.01 Å)	^c^Δ σ^2^(Å^2^)	R factor
**Silver species**					
Ag	Ag-Ag	4.32	2.66	0.01012	0.35801
Ag_2_O	Ag(I)-O	1.00	2.06	0.00092	0.00141
AgO	Ag(II)-O	0.95	2.05	0.00059	0.00143
Ag_2_S	Ag(I)-S	3.08	2.33	0.01361	0.00441

## Discussion

In this study, we found that Ag-NP concentrations exceeding 5 ppm were capable of inhibiting *B*. *subtilis* growth in rich medium (Fig 2A), while concentrations of just 0.1 ppm was sufficient to halt growth in minimal media cultures of *B*. *subtilis* (Fig 2B). At Ag-NP concentrations of 10 ppm and above, a 2 log reduction in bacterial colony-forming units was observed (Fig 2C). These results are in line with previous observations made by Kim *et al*., who found that Ag NPs averaging 10 nm in diameter were able to inhibit *B*. *subtilis* growth, inducing a belated log phase at concentrations of 5 ppm [59]. Interestingly, we found that 0.1 ppm concentrations of Ag NPs was able to halt *B*. *subtilis* growth in minimal media, and this may explain the 35% reduction in microbial mass observed for soil samples treated with 0.14 mg Ag/kg soil in a recent field scenario study [43]. In addition, a previous study found that 100 ppm concentrations of 50-nm Ag NPs led to a 2 log reduction in *B*. *subtilis* cell numbers, an observation that supports our findings and remains consistent with the understanding that smaller nanoparticles are more toxic to cells and can therefore inhibit cell growth at lower concentrations [35]. However, a recent study showed that 3-nm Ag NPs were only able to inhibit *B*. *subtilis* growth at concentrations of 20 ppm [36], which is puzzling as these Ag NPs were clearly smaller than 10 nm, but did not demonstrate greater toxicity or inhibitory effects. This suggests that the shape of Ag NPs may also be an important factor in determining toxicity, as shape and structure could potentially affect the ability of Ag NPs to dissolve in liquids or release Ag^+^ ions [18]. In addition, it is known that the size and shape of Ag NPs can affect their plasmonic properties [29, 31], which in turn may have bearing on the physical and biological activity of these nanoparticles. Further research will be needed to assess whether changes in surface plasmon resonance can modulate Ag-NP antimicrobial activity. Furthermore, it has been reported that in biological fluids such as blood, the plasmonic properties and high surface energy of nanoparticles can attract proteins to form a coating, known as the protein corona [60–61]. The protein corona may affect the physical, chemical, and biological properties of nanoparticles in ways that are yet to be understood; moreover, the composition of proteins in the corona varies between nanoparticles and evolves dynamically over time, making it difficult to pinpoint any specific corona effects [61]. Compared to blood or lymphatic fluid, bacterial culture medium may be less complex, but in future experiments, it would be interesting to assess the presence and effect of protein coronas upon Ag NPs in bacterial cultures. More importantly, further research may be needed to understand how protein coronas could affect the impact of Ag NPs inadvertently released into soil or water.

Previous research has shown that Ag NPs can cause membrane damage in Gram-negative *Escherichia coli* [62], but the results were derived from energy filtered TEM analysis, a technique known to induce slight membrane damage during sample preparation and analysis. In light of this, we elected to use *in vivo* PI staining to confirm if Ag NPs could disrupt bacterial cell membranes in *B*. *subtilis*. To our knowledge, this is the first study to examine the effect of Ag-NPs on bacterial membrane integrity using the PI assay. Our findings revealed that at Ag-NP concentrations ≥ 25 ppm, the number of cells with membrane damage increased (Fig 3B), and further indicated that membrane degradation occurred as a result of increased permeability induced by Ag NPs. It is possible that Ag-NP treatment damaged cellular membranes to increase permeability, or penetrated bacterial cell membranes to cause protein dysfunction. Our results further showed that reductase activity (Fig 3A) and P_hag_-GFP expression levels (Fig 3C and 3D) were lowered by Ag NPs, suggesting that decreased protein expression and/or an enhanced ROS response may also be involved in loss of membrane integrity and cell viability.

There is an ongoing debate over whether Ag NPs inherently possess toxicity, or if microbial toxicity is dependent on leached Ag^+^ ions from nanoparticles. To clarify this issue, we utilized XANES/EXAFS analysis to assess the fine structure of silver particles present within *B*. *subtilis* cells treated with 100 ppm of Ag NPs. Following comparison with the spectra of silver standards (Ag, AgO, Ag_2_O, and Ag_2_S), we found that Ag_2_O best fits the spectra derived from Ag NP-treated *B*. *subtilis* cells (Figs 4 and 5A–5E, and Table 1). This data strongly suggests that in aqueous solutions, Ag NPs leach off Ag^+^ ions, which subsequently enter bacterial cells and are oxidized to form Ag_2_O. If Ag NPs entered cells directly, it would have been possible to detect spectra corresponding to the Ag standard. To the best of our understanding, this is the first study to directly analyze silver particles present within bacterial cells treated with Ag NPs, and the results indicate that Ag^+^ ions are primarily responsible for Ag-NP microbial toxicity.

In summary, our results support the theory that Ag NPs exert microbial toxicity through the release of Ag^+^ ions that subsequently penetrate into bacterial cells, and to the best of our knowledge, this is the first study to provide direct evidence of this through XANES/EXAFS analyses. We further show that Ag NPs can inhibit the growth of Gram-positive *B*. *subtilis* bacteria, and exert toxicity by damaging cellular membranes, degrading chromosomal DNA, lowering reductase activity, and reducing protein expression. These results highlight the risks of engineered nanoparticle contaminants to soil and water ecosystems, and suggest that further research is needed to better understand the potential impact of Ag-NP contamination.

## Supporting Information

S1 File Supporting information. (A) Agarose gel electrophoresis analysis of Ag-NP effects on *B*. *subtilis* chromosomal DNA. (B) TEM images of *B*. *subtilis* cells subjected to Ag-NP treatment. (C) Elemental analysis of *B*. *subtilis* cells treated with 50, 100, or 200 ppm of Ag NPs.
(DOCX)
