# Supplementary material for: The Antimicrobial Properties of Silver Nanoparticles in Bacillus subtilis Are Mediated by Released Ag+ Ions
Source: PLoS One. 2015 Dec 15;10(12):e0144306. doi: 10.1371/journal.pone.0144306 (PMC4682921; doi:10.1371/journal.pone.0144306)
Supplement: S1 File — (A) Agarose gel electrophoresis analysis of Ag-NP effects on B. subtilis chromosomal DNA. (B) TEM images of B. subtilis cells subjected to Ag-NP treatment. (C) Elemental analysis of B. subtilis cells treated with 50, 100, or 200 ppm of Ag NPs. (DOCX) [file pone.0144306.s001.docx]

**The Antimicrobial Properties of Silver Nanoparticles in *Bacillus subtilis* are Mediated by Released Ag^+^ Ions**

**Supporting Information**

**S1 File. Supporting information.**

**(A) Agarose gel electrophoresis analysis of Ag-NP effects on *B. subtilis* chromosomal DNA.**


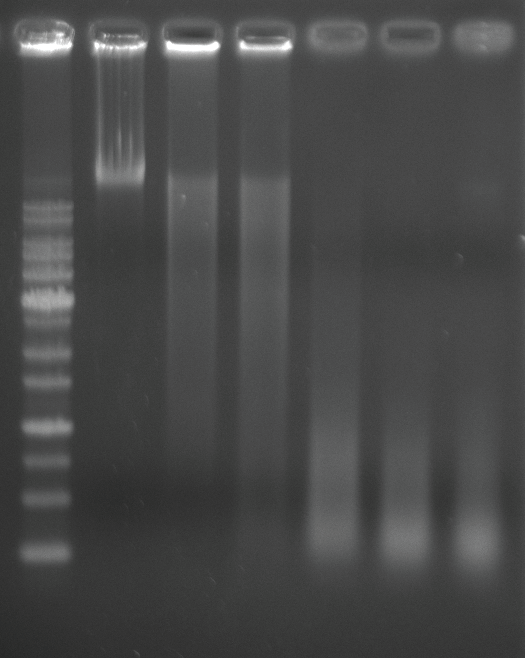


**M 0 1 5 10 25 50 (ppm)**

**(B) TEM images of *B. subtilis* cells subjected to Ag-NP treatment.**


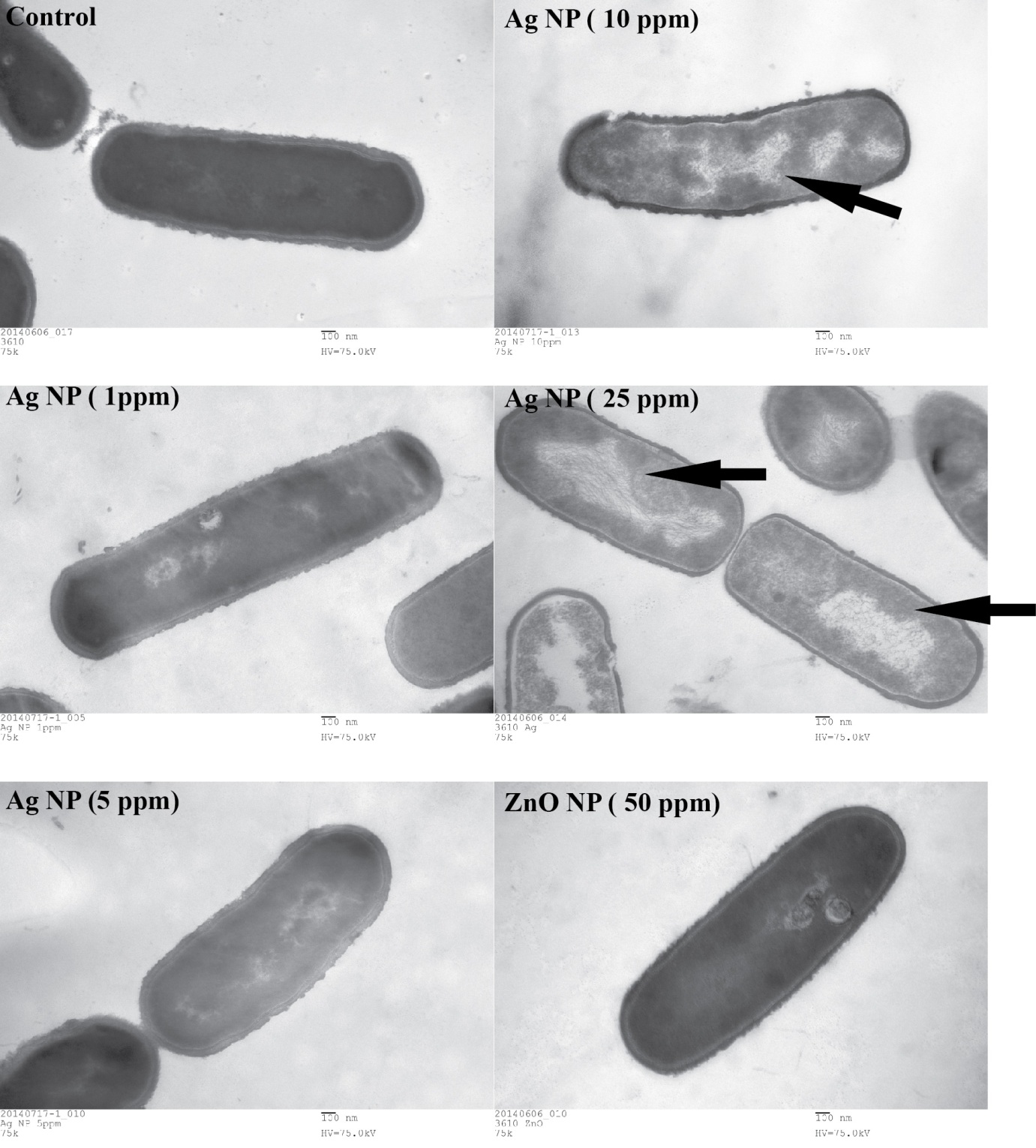


**(C) Elemental analysis of *B. subtilis* cells treated with 50, 100, or 200 ppm of Ag NPs.**

| Sample | Element (%) | | | | |
| --- | --- | --- | --- | --- | --- |
|  | N | C | S | H | O |
| 50 ppm | 11.45 | 48.87 | N.D. | 13.03 | 6.65 |
| 100 ppm | 15.90 | 46.82 | N.D. | 21.10 | 3.18 |
| 200 ppm | 23.17 | 43.03 | N.D. | 6.81 | 6.99 |

N.D.: Not detectable.
